# General diseases and medications in 687 patients reporting on adverse effects from dental materials

**DOI:** 10.1007/s00784-023-05064-5

**Published:** 2023-05-22

**Authors:** Fabian Cieplik, Karl-Anton Hiller, Konstantin J. Scholz, Gottfried Schmalz, Wolfgang Buchalla, Pauline Mittermüller

**Affiliations:** 1 Department of Conservative Dentistry and Periodontology, University Hospital Regensburg, Franz-Josef-Strauß-Allee 11, 93053 Regensburg, Germany; 2 Department of Periodontology, School of Dental Medicine, University of Bern, Bern, Switzerland

**Keywords:** Dental materials, Adverse effects, Burning mouth, Taste disorders, Dry mouth, Allergy, Medication, General disease

## Abstract

**Objectives:**

Examination of patients claiming adverse effects from dental materials can be very challenging. Particularly, systemic aspects must be considered besides dental and orofacial diseases and allergies. Therefore, the aim of this study was to investigate a cohort of 687 patients reporting on adverse effects from dental materials focusing on findings related to known general diseases or conditions or medication-related findings with relevance to their subjective complaints.

**Methods:**

Six hundred eighty-seven patients visiting a specialized consultation on claimed adverse effects from dental materials were retrospectively investigated for their subjective complaints, findings related to known general diseases or conditions, medication-related findings, dental and orofacial findings, or allergies with relevance to their subjective complaints.

**Results:**

The most frequent subjective complaints were burning mouth (44.1%), taste disorders (28.5%), and dry mouth (23.7%). In 58.4% of the patients, dental and orofacial findings relevant to their complaints could be found. Findings related to known general diseases or conditions or medication-related findings were found in 28.7% or 21.0% of the patients, respectively. Regarding medications, findings related to antihypertensives (10.0%) and psychotropic drugs (5.7%) were found most frequently. Relevant diagnosed allergies toward dental materials were found in 11.9%, hyposalivation in 9.6% of the patients. In 15.1% of the patients, no objectifiable causes for the expressed complaints could be found.

**Conclusions:**

For patients complaining of adverse effects from dental materials, findings related to known general diseases or conditions and medications should be given particular consideration, while still in some patients, no objectifiable causes for their complaints can be found.

**Clinical relevance:**

For patients complaining about adverse effects from dental materials, specialized consultations and close collaboration with experts from other medical fields are eligible.

## Introduction

In modern dentistry, a wide variety of dental materials with different requirements in terms of esthetic and mechanical properties are used to restore lost tooth structures or to replace teeth, intended to remain in the patients’ oral cavities and in direct contact with oral tissues for many years or decades [1, 2]. This can inevitably lead to the occurrence of adverse effects from these materials, as has been reported in the literature [3–15].

However, the general prevalence of adverse effects from dental materials is considered quite low [3–5, 13–17]. For instance, Jacobsen and Hensten-Petterson found in a series of studies that the prevalence of adverse effects from dental materials was about one case per year per periodontologist [4], about 1% for patients in orthodontics [3], and about 0.33% for patients in prosthodontics [5]. Kallus and Mjör reported adverse effects from dental materials used in the treatment of 13,325 patients in 15,820 appointments in 46 cases (0.35%) [14]. Van Noort et al. summarized that the Norwegian, Swedish, and UK projects for systemic monitoring of adverse reactions to dental materials had received 1268 reports over 11 years, 848 reports over 5.5 years, or 1117 reports over 3 years, respectively, till their publication in 2004 [16].

In December 1998, a specialized consultation for suspected adverse effects from dental materials was established in the Department of Conservative Dentistry and Periodontology of the University Hospital Regensburg [10, 11], resulting from a former study on patients complaining about adverse effects from dental alloys (except amalgams) conducted from 1995 till 1997 [8]. Until September 2021, 687 patients have been examined and diagnosed clinically by one single general dentist with special interest and experience in adverse effects from dental materials (Pauline Mittermüller, née Garhammer). Two retrospective studies have investigated this patient cohort so far [10, 11].

The first study summarized data from the first 500 patients from this cohort (examined and diagnosed from December 1998 to early 2015) with a focus on allergies and found that allergies to dental materials or their components contributed to the subjective complaints expressed by the patients in only 14% of the cases [10]. The second study on 625 patients from this cohort (examined and diagnosed to mid 2019) therefore investigated non-allergy-related dental and orofacial findings relevant to the complaints expressed by the patients [11]. Here, it was found that dental and orofacial findings occurred more frequently than allergies as possible causes for the subjective complaints expressed by the patients, but still 28% of the patients exhibited neither an allergy nor a dental or orofacial finding with potential relevance for their complaints [11]. This raises the question which other factors may be causative for the complaints in this group of patients claiming adverse effects from dental materials.

Therefore, the aim of the present study was to investigate an enlarged cohort of 687 patients reporting on adverse effects from dental materials with special focus on findings related to known general diseases and medications that could be deemed relevant for their subjective complaints.

## Material and methods

### Study design

All patients visiting the specialized consultation for suspected adverse effects from dental materials in the Department of Conservative Dentistry and Periodontology of the University Hospital Regensburg between December 1998 and September 2021 were included in the present study. No further inclusion or exclusion criteria were applied. These 687 patients either came on their own initiative, or they were referred to this specialized consultation by dentists from the Eastern Bavarian region (Niederbayern and Oberpfalz), which comprises about two million inhabitants. All data of these patients were retrieved retrospectively and processed anonymized ensuring that no allocation of data to the identity of an individual patient was possible. Therefore, no approval of an institutional review board was required.

### Medical anamnesis and clinical examinations

Medical history, medications, and allergies were asked from the patients in a standardized manner. First, general (general health) and specific (oral health) anamneses were taken, including information on type, location, time of appearance, and duration of the subjective complaints expressed by the patients. Second, thorough extraoral and intraoral examinations and photo documentations were taken. X-ray examinations (mainly orthopantomograms and dental films) were performed in case of justifying medical indications only.

Allergies as well as dental and orofacial findings were not the focus of the present study but are reported for the sake of completeness. The related methods are reported in brief in the following, but the reader may be kindly referred for detailed methods to [10] with regard to allergy testing and [11] with regard to dental and orofacial examinations. Allergies were tested by means of patch testing. Margins of fixed dental prostheses (FDPs) were evaluated using dental explorers and were defined as insufficient if they could be probed (dichotomic decisions based on Felton et al. [18]). Removable dental prostheses (RDPs) were examined for mechanical irritations (e.g., insufficient hold, swaying upon pressure, or occurrence of irritating ridges or pressure bruises). All FDPs and RDPs were also examined for manufacturing faults (e.g., corrosion spots, shrink holes, solder points, perforations). Manufacturing faults or mechanical irritations were deemed relevant in the case of close spatial and temporal connection to the subjective complaints expressed by the patients. Clinical functional analyses were carried out according to the condensed temporomandibular disorders screening by Krogh-Poulsen [19, 20], comprising clinical examination and interview of the patients with respect to oral parafunctional habits, bruxism, pressing and grinding of teeth, abraded dentition, pain on palpation of the masticatory muscles, clicking or pain in the temporomandibular joint, etc. Clinical functional diagnoses were considered relevant in the case of close spatial and temporal connection to the subjective complaints expressed by the patients. The gingiva, oral mucosa, and tongue were examined for changes or pathologies related to orofacial diseases (e.g., oral lichen planus, leukoplakia, anomalies of the tongue). Orofacial diseases were deemed relevant in the case of close spatial and temporal connection to the occurrence of the subjective complaints expressed by the patients. The salivary flow rate (SFR) was measured by letting the patients collect gum-stimulated whole saliva for a period of 5 min. Stimulated SFR of ≤ 0.7 mL/min was considered hyposalivation [21]. The level of oral hygiene was assessed by means of the full-mouth Papilla Bleeding Index (PBI) as described by Saxer and Mühlemann [22]. All clinical examinations were performed by one experienced dentist (PM) with clinical experience of more than 25 years in examining and diagnosing patients claiming adverse effects from dental materials.

Findings related to anamnestically known general diseases and conditions, or medication-related findings, were considered relevant if they were in close temporal connection to the subjective complaints specified by the patients and could be regarded as causative for these subjective complaints, e.g., as concomitant symptoms of general diseases or conditions, or as known side effects of medications.

### Data analysis

Full-mouth PBI data are given as medians including 1^st^ and 3^rd^ quartiles. All other data are presented descriptively as frequency tables. All calculations were performed using SPSS, v. 25 (SPSS Inc., Chicago, IL, USA).

## Results

### Patient characteristics and oral hygiene level

The median (1^st^; 3^rd^ quartile) age of all 687 patients included in this study was 58 (50; 66) years. From these 687 patients, there were 555 females (80.8%) and 132 males (19.2%). The median (1^st^; 3^rd^ quartile) full-mouth PBI was found to be 39.7% (28.7%; 51.7%) and could be examined in 638 (92.9%) out of the 687 patients. In the other 49 patients, PBI measurements were not possible due to edentulism, refusal of measurements, or suffering from diseases (e.g., valvular transplants) militating against PBI measurements without preventive systemic antibiotics.

### Subjective complaints

The most often reported subjective complaints in this cohort of 687 patients were burning mouth (44.1%), taste irritations (i.e., metal, sour, bitter, salty, sweet, or reduced taste; 28.5%), and dry mouth (23.7%), as shown in Table 1. The list of subjective complaints expressed by less than 3% of the patients comprised 120 entries, such as speech impairment, problems with the eyes, alopecia, nervousness, anxiety states, or forgetfulness. Ninety-five patients (13.8%) reported one subjective complaint, 166 (24.2%) two, 137 (19.9%) three, 119 (17.3%) four, and 154 patients (22.0%) reported five or more (up to 12) subjective complaints. Furthermore, 16 patients (2.3%) did not report on any subjective complaint, mostly because of abatement of the complaints before their appointment in our specialized consultation.

**Table 1:** Subjective complaints reported by the 687 patients

Subjective complaint^1^	Frequency (%)^2^
No subjective complaint	2.3
Burning mouth	44.1
Taste disorders (metal, sour, bitter, salty, sweet, reduced taste)	28.5
Dry mouth	23.7
Toothache/jaw pain	19.8
Gingivitis	16.2
Paresthesia	14.0
Weakness	8.4
Gingival bleeding	6.8
Headache/migraine	6.1
Swelling	5.7
Intestinal problems	5.4
Poor denture retention	5.4
Sensation of pressure	5.4
Electrical sensations	5.1
Painful swallowing/sore throat	5.1
Gingival pain	4.8
Red palate	4.7
Articular pain	4.1
Itching	3.8
Red/inflamed tongue	3.8
Dry lips	3.3
Blisters	3.1
Facial pain	3.1
Reduced ability for chewing	3.1

^1^Subjective complaints reported by at least 3% of the 687 patients are listed

^2^100% = 687 patients, multiple entries per patient were possible. Sixteen patients (2.3%) did not report any subjective complaint. Ninety-five patients (13.8%) reported one subjective complaint, 165 (24.0%) two, 138 (20.1%) three, 119 (17.3%) four, and 154 patients (22.4%) reported five or more (up to 12) subjective complaints

### Findings with relevance for the subjective complaints expressed by the patients

Table 2 summarizes the findings that were considered relevant for the subjective complaints expressed by the patients. Relevant dental and orofacial findings could be diagnosed in 401 patients (58.4%). Relevant findings related to known general diseases were detected in 197 patients (28.7%) and relevant medication-related findings in 144 patients (21.0%). Allergies with relevance for the subjective complaints were diagnosed in 82 patients (11.9%), and hyposalivation was found in 67 patients (9.6%). In 104 patients (15.1%), nothing could be diagnosed that could be deemed relevant for the complaints expressed by the patients.

**Table 2:** Findings from the 687 patients with clinical relevance for their subjective complaints

Relevant finding^1^	Frequency (%)^2^				
	All^a^	Burning mouth^b^	Taste disorders^c^	Dry mouth^d^	Toothache/jaw pain^e^
No relevant finding	15.1	26.6	12.8	6.8	14.7
Dental and orofacial findings^3^	58.4	63.7	59.7	64.4	68.4
Findings related to known general diseases or conditions^4^	28.7	35.6	31.6	35.6	24.3
Medication-related findings^5^	21.0	25.1	25.5	43.6	19.1
Allergies	11.9	7.9	11.7	6.1	5.9
Hyposalivation	9.6	12.9	10.2	33.7	6.6

^1^All relevant findings from the 687 patients are listed

^2^Multiple entries per patient were possible. One hundred four patients exhibited no relevant finding (15.1%). Three hundred fifty-seven patients (52.0%) had one relevant finding, 160 patients (23.3%) had two, 51 patients (7.4%) three, and 15 patients (2.2%) four relevant findings

^3^A refined evaluation can be found in Table 3

^4^A refined evaluation can be found in Table 4

^5^A refined evaluation can be found in Table 5

^a^100% = all 687 patients

^b^100% = 303 patients complaining about burning mouth (44.1% of the 687 patients)

^c^100% = 196 patients complaining about taste irritations (28.5% of the 687 patients)

^d^100% = 163 patients complaining about dry mouth (23.7% of the 687 patients)

^e^100% = 136 patients complaining about toothache/jaw pain (19.8% of the 687 patients)

Table 2 further summarizes the findings considered relevant for patients complaining about burning mouth, taste disorders, dry mouth, or toothache/jaw pain, respectively, which were found to be the four most frequently reported subjective complaints (see Table 1).

#### Dental and orofacial findings

Two hundred thirty-three out of the 401 patients (58.1%) with relevant dental and orofacial findings exhibited no other relevant finding, while 168 patients (41.9%) were diagnosed with at least one other relevant finding (mostly findings related to known general diseases or conditions or medication-related findings). Table 3 summarizes all relevant dental and orofacial findings with functional symptoms (22.0%), orofacial diseases (17.5%), and mechanical irritations (14.4%) being found most frequently. More details on this aspect have been presented considering a sub-cohort of 625 patients [11].

**Table 3:** Dental and orofacial findings in the 687 patients with clinical relevance for their subjective complaints

Relevant dental or orofacial finding^1^	Frequency (%)^2^
No relevant dental or orofacial finding	41.6
Functional symptoms (e.g., oral parafunctional habits, bruxism, pain of the masticatory muscles or in the temporomandibular joint)	22.0
Orofacial diseases (e.g., anomalies of the tongue, oral lichen planus, lichenoid contact reactions)	17.5
Mechanical irritations (e.g., insufficient margins of fixed dental prostheses or insufficient hold or swaying upon pressure of removable dental prostheses)	14.4
Plaque-related symptoms (e.g., gingivitis)	10.6
Tooth-related symptoms (e.g., dental caries, tooth or root fractures, periodontal defects)	9.9
Manufacturing faults (e.g., corrosion spots, shrink holes, solder points or perforations)	6.6

^1^All relevant dental and orofacial findings of the 687 patients are listed

^2^100% = 687 patients, multiple entries per patient were possible

For more details, the reader may be kindly referred to the previous publication focusing on dental and orofacial findings with clinical relevance for the subjective complaints expressed by 625 patients claiming adverse effects from dental materials [11]

#### Findings related to known general diseases or conditions

One hundred fifty patients (76.1%) had one and 47 (23.9%) at least two relevant findings related to known general diseases or conditions. Out of these 197 patients, 47 (23.9%), had no further relevant finding, while 150 patients (76.1%) were diagnosed with at least one other relevant finding (mostly dental and orofacial findings or medication-related findings).

**Fig. 1:**
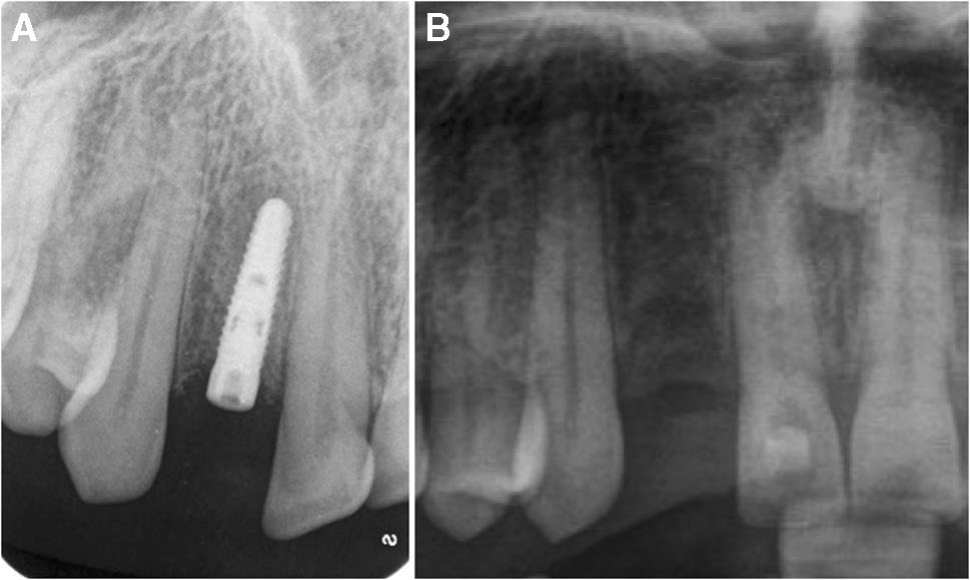
Clinical example for a relevant finding related to mental and behavioral disorders. Patient with throbbing pain after implant placement in regio 12 (**A**) and subsequent removal of the implant in regio 12 and root canal treatment in tooth 11 (**B**) due to spreading pain. Referral to neurologist/psychiatrist revealed pain disorder, atypical odontalgia, and depression

**Fig. 2:**
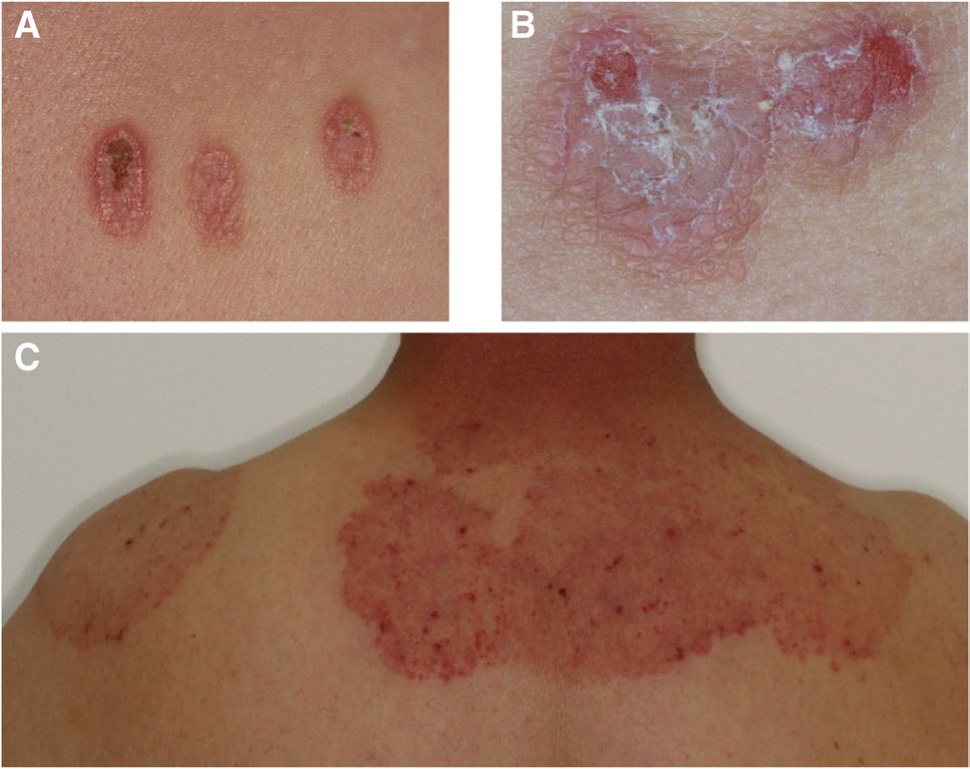
Clinical examples for relevant findings related to diseases of the skin and subcutaneous tissue. **A** Patient with skin florescences on the arms and back, stating that symptoms occurred in close temporal relation to insertion of a dental prosthesis. Consultation in dermatology revealed Prurigo simplex subacuta with known history of diabetes mellitus type II. **B** Patient with skin florescences on arms, legs, and back after insertion of new fixed dental prostheses. Referral to dermatologist revealed cutaneous lichen planus. **C** Patient complaining about skin problems on chest and back for several years. Condition prior to extensive dental rehabilitation with uncertainty regarding selection of dental materials. Referral to dermatologist revealed fungal disease of the skin

**Fig. 3:**
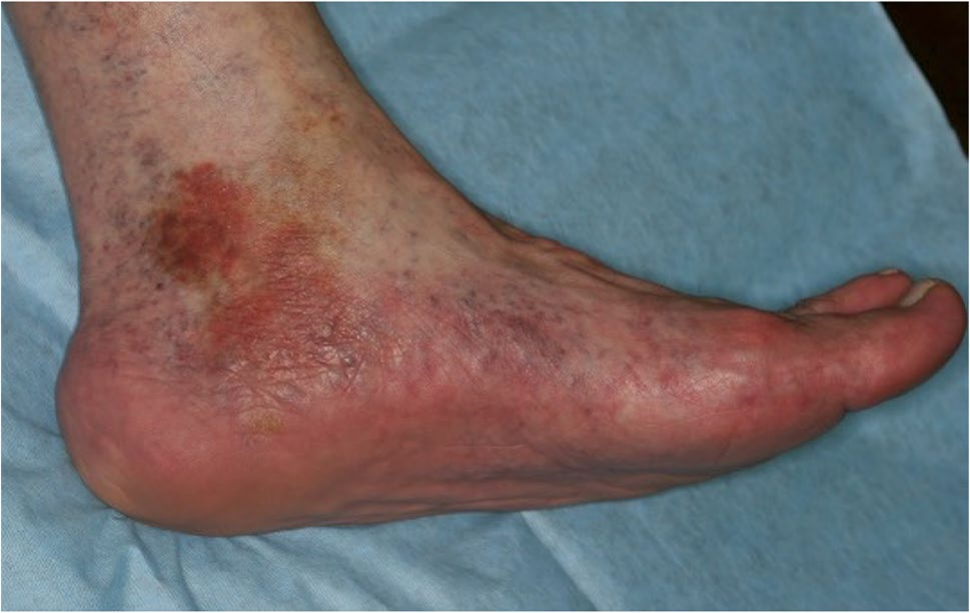
Clinical example for a relevant finding related to diseases of veins. Patient presents with skin lesions on the feet and suspects adverse effects from dental materials. Referral to dermatologist revealed chronic venous insufficiency

Table 4 summarizes all relevant findings related to known general diseases or conditions, whereby mental and behavioral disorders (11.9%), diabetes mellitus (3.1%), disorders of the thyroid gland (2.5%), and diseases of the musculoskeletal system and connective tissue (2.5%) were found most frequently. Figures 1, 2, and 3 show clinical examples for relevant findings related to general diseases or conditions.

**Table 4:** Findings related to known general diseases or conditions in the 687 patients with clinical relevance for their subjective complaints

Relevant finding related to known general diseases or conditions (ICD-10 code)^1^	Frequency (%)^2^
No relevant finding related to known general diseases or conditions	71.3
Mental and behavioral disorders (F00–F99)	11.9
Diabetes mellitus (E10–E14)	3.1
Disorders of thyroid gland (E00–E07)	2.5
Diseases of the musculoskeletal system and connective tissue (M00–M99)	2.5
Diseases of the digestive system (K00–K93)	2.2
Personal history of malignant neoplasm (Z85)	2.0
Iron deficiency anemia (D50)	1.7
Diseases of the nervous system (G00–G99)	1.7
Heartburn (R12)	1.5
Sleep disorders (F51 / G47)	1.2
Diseases of the skin and subcutaneous tissue (L00–L99)	1.0
Headache/migraine (R51/G43)	0.7
Lyme disease (A68 / A69)	0.6
Herpesviral [herpes simplex] infections (B00), Zoster [herpes zoster] (B02)	0.4
Vitamin deficiency (E50–E64)	0.4

^1^Relevant findings related to known general diseases or conditions in at least 3 patients (0.4% of the 687 patients) are listed

^2^100% = 687 patients, multiple entries per patient were possible. Four hundred ninety patients (71.3%) exhibited no relevant finding related to known general diseases or conditions. One hundred fifty patients (21.8%) had one relevant finding, 38 patients (5.5%) had two, 6 patients (0.9%) three, and 3 patients (0.4%) four relevant findings

#### Medication-related findings

Ninety-four from the 144 patients (65.3%) with relevant medication-related findings exhibited just one and 50 (34.7%) at least two medication-related findings. Furthermore, 23 from those 144 patients (16.0%), had no further relevant finding, while 121 patients (84.0%) were diagnosed with at least one other relevant finding (mostly dental and orofacial findings, findings related to known general diseases or conditions, or hyposalivation).

**Fig. 4:**
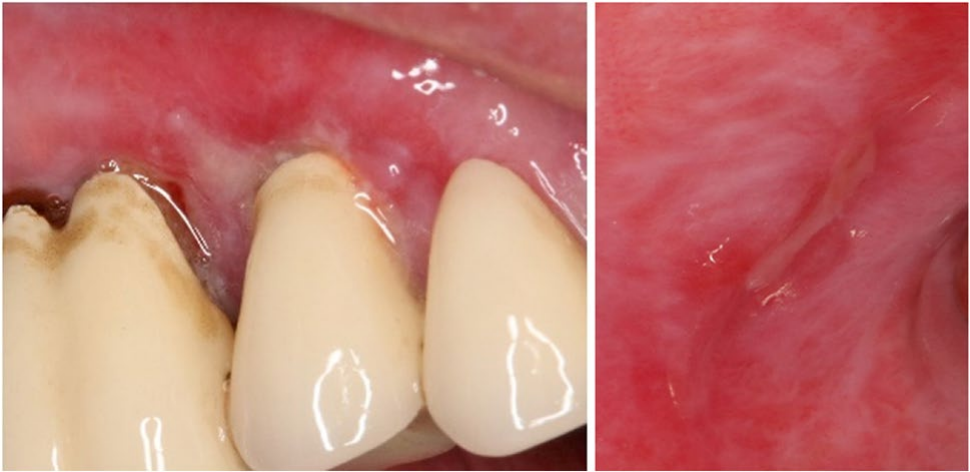
Clinical example for a relevant medication-related finding. Patient reports recurring painful mucosal lesions and aphthae. Histological examination of two biopsies showed a chronic ulcer and chronic gingivitis (without any evidence of malignancy). Medical history revealed that the patient is taking the angiotensin-converting-enzyme (ACE) inhibitor ramipril, which has oral ulcers, aphthae, and aphthous stomatitis as known side effects

Table 5 summarizes all relevant medication-related findings, whereby findings related to antihypertensives such as beta-blockers, calcium antagonists and renin-angiotensin system inhibitors (10.0%), psychotropic drugs (5.7%), and antilipidemics (2.3%) were found most frequently. Figure 4 shows a clinical example for a relevant medication-related finding.

**Table 5:** Findings related to medications of the 687 patients with clinical relevance for their subjective complaints

Relevant medication-related finding^1^	Frequency (%)^2^
No relevant medication-related finding	79.0
Antihypertensives (e.g., beta-blockers, calcium antagonists, renin-angiotensin system inhibitors)	10.0
Psychotropic drugs (antidepressants, antipsychotics)	5.7
Antilipidemics (statins)	2.3
Analgesics, non-steroidal anti-inflammatory drugs (NSAIDs)	1.3
Broncholytic anti-asthmatic drugs	1.3
Gastrointestinal drugs	1.3
Diuretics	1.2
Urologic drugs	1.0
Gout drugs	0.7
Hypnotics, sedatives	0.7
Sexual hormones and their inhibitors	0.7
Antidiabetics	0.4
Anticonvulsants	0.4
Parkinson’s disease drugs and other drugs for extrapyramidal disorders	0.4

^1^Relevant medication-related findings in at least 3 patients (0.4% of the 687 patients) are listed

^2^100% = 687 patients, multiple entries per patient were possible. Five hundred forty-three patients (79.0%) exhibited no medication-related finding. Ninety-four patients (13.7%) had one relevant finding, 36 patients (5.2%) had two, 10 patients (1.5%) three, 3 patients (0.4%) four, and one patient (0.1%) five relevant findings

#### Allergies

Out of the 82 patients with relevant allergies toward a dental material allergen, 48 patients (58.5%) exhibited no further relevant finding, while in 34 patients (41.5%), at least one other relevant finding could be diagnosed (mostly findings related to known general diseases or conditions). This aspect was presented in detail earlier considering a sub-cohort of 500 patients [10], and has also been covered considering the sub-cohort of 625 patients [11].

#### Hyposalivation

Out of the 66 patients diagnosed with hyposalivation, 6 patients (9.1%) had no other relevant finding, while 60 patients (90.9%) exhibited at least one other relevant finding (dental and orofacial findings, findings related to known general diseases or conditions, or medication-related findings).

## Discussion

### Study design and study population

The present study is a retrospective cohort study investigating all patients visiting a specialized consultation for suspected adverse effects from dental materials without applying further inclusion or exclusion criteria. While our previous works on subpopulations from this cohort focused on allergies [10] or dental and orofacial findings [11], respectively, the present study on 687 patients investigated findings related to known general diseases or conditions as well as medications that could be deemed relevant for the subjective complaints expressed by the patients. Details on the study population, such as age and gender distribution, have been extensively discussed in our previous studies [10, 11]. The demographic results (strong female predominance and median age of 58 years) are in accordance with our previous works [8, 10, 11] as well as studies on other cohorts of patients claiming adverse effects from dental materials [16, 17, 23, 24]. The overall oral hygiene level as measured by the PBI (median 39.7%) can be considered acceptable [25].

### Subjective complaints

The patients visiting this specialized consultation described a wide variety of different local or general complaints which they attributed to be adverse effects from dental materials, in line with literature data from similar patient cohorts [17, 24, 26]. Patients often report multiple subjective complaints like up to 12 in our cohort, which makes clinical appraisal of these complaints very challenging, further complicated by the fact that these complaints may not be related to dental materials at all [10, 11]. For instance, for toothache/jaw pain, which made up the 4^th^ most reported complaint and at first glance appears to be easily objectifiable by dental or orofacial findings, relevant dental or orofacial diseases could be diagnosed in only 68.4% of the patients (Table 2). This situation gets even more complex when considering the three most often reported complaints in this cohort, burning mouth, taste disorders, and dry mouth, which can be caused by a wide range of etiologies, including general diseases or conditions or as side effects from medications [27–32]. This underscores that general dentists should consider a wide range of possible explanations (see also below) when examining a patient who complains of adverse effects from dental materials.

### Findings with relevance for the complaints expressed by the patients

#### Dental and orofacial findings and allergies

Dental and orofacial findings were found to relevantly contribute to the subjective complaints expressed by the patients in a majority of the cases (58.4%), mostly due to orofacial symptoms like oral lichen planus or anomalies of the tongue or due to functional symptoms such as habits or bruxism, which has been discussed in detail previously [11]. Regarding the latter, it must however be considered that the condensed temporomandibular disorders screening applied in the present study [19, 20] has been discussed critically nowadays [33], which has however also been considered in the interpretation of the findings, as discussed in our previous study [11].

Also consistent with our previous studies [8, 10, 11], only 11.9% of patients were found to have allergies that could be considered relevant to the patients’ symptoms. For more details on the role of allergies along with the patch testing method and the most common allergens, the reader may be referred to our previous publication [10].

#### Findings related to known general diseases or conditions

In the present cohort of 687 patients, findings related to known general diseases or conditions could be considered relevant to the complaints expressed by the patients in more than one quarter of the cases. However, most of the patients just had one general disease or condition with relevance for these complaints. Some of the relevant general diseases or conditions found in this cohort have also been described to be among the leading contributors to global burden of disease in people aged 60 years and older in 2010, such as malignant neoplasies, musculoskeletal diseases, mental and neurological disorders, diabetes mellitus, and digestive diseases [34]. It is known that systemic diseases can manifest orally and that these oral manifestations can be the first, only, or the most distressing symptoms of the underlying disease [35]. For instance, diabetes mellitus can lead to dry mouth, taste disorders, or even a loss of sensation due to diabetic neuropathy [30, 35]. Inflammatory bowel diseases, particularly Crohn’s disease, can lead to either specific (e.g., cobblestoning mucosa) or unspecific oral manifestations, such as aphthous or lichenoid lesions, cheilitis angularis, or taste alterations [36, 37]. Thyroid disorders such as hypothyroidism or hyperthyroidism can manifest in taste disorders and glossitis or burning mouth, respectively [38], and iron deficiency anemia usually is associated with burning mouth and dry mouth [39]. Accordingly, the proportions of patients with relevant findings related to general diseases was higher in the subgroups complaining about burning mouth, dry mouth, or taste disorders as compared to the whole cohort of 687 patients (Table 2).

The situation becomes even more complex for the group of mental and behavioral disorders that were not diagnosed by the investigator but were known from the patient’s medical history and were considered relevant to the expressed complaints in about 12% of cases, which is slightly above the age-standardized general prevalence for mental disorders of 10.3% described for central Europe in 2019 [40]. For example, there is ongoing debate on the role of specific psychiatric features in burning mouth syndrome [41–43]. On the one hand, some psychiatric features such as anxious traits, personality disorders, or somatization have been discussed to play a role in pathogenesis of burning mouth syndrome (BMS), while on the other hand, suffering from BMS may lead to secondary psychiatric symptoms [41–43]. In addition, it is discussed that structural and functional deficits in the nervous system, especially circadian rhythm disturbances, are involved in the pathogenesis of BMS [44]. Likewise, oral functional habits such as tongue pressing can cause BMS-like symptoms [28], as discussed in more detail in our previous work [11]. Furthermore, depressions, anxious disorders, and somatization (also in terms of the so-called environmental somatization syndrome) may be particularly relevant in patients complaining about adverse effects from dental materials as shown for example in several studies focusing on dental amalgams [26, 45–48]. Furthermore, depressions may also be associated with pain disorders such as atypical odontalgia [49, 50], as also shown in the clinical example in Fig. 2. For instance, Takenoshita et al. reported that about 60% of their patients suffering from atypical odontalgia had been diagnosed with some psychiatric disease [50]. Interestingly, pain disorders that include persistent and chronic pain seem to be more common in the head and neck than in any other part of the body [51].

#### Medication-related findings

Many medications can induce adverse reactions or unintended side effects in the oral cavity [31, 32] which may be misinterpreted as adverse effects from dental materials. Here, we retrieved potential medication-related findings from the medical package inserts of the medications reported by the patients [52], and found medication-related findings with relevance for the patients’ complaints in 21% of the cases. The main oral symptoms that usually are associated with medications are dry mouth and taste disorders [31, 32, 52–55]. Accordingly, patients complaining about dry mouth exhibited twice as often medication-related findings as compared to the whole cohort of 687 patients (Table 2). Furthermore, hyposalivation was recorded about three times more often in patients complaining about dry mouth (Table 2), although it is also associated with BMS-like symptoms or taste disorders and thus negatively affects oral health-related quality of life [55, 56].

Particularly, psychotropic drugs like antidepressants (e.g., serotonin agonists or serotonin re-uptake blockers) and antipsychotics (e.g., phenothiazines) or antihypertensives have dry mouth or xerostomia as common side effects [31, 32, 52]. On the other hand, taste disorders are associated with the intake of antihypertensives such as angiotensin-converting-enzyme (ACE) inhibitors or antilipidemics such as statins [31, 53, 54]. With increasing age, the number of medications increases and with it the risk for medication-related side effects such as dry mouth, hyposalivation, or taste disorders [52, 55]. Considering demographic trends and aging of society, increasing medication use and polypharmacy may lead to an increasing prevalence of such side effects [55].

## Conclusions

A synopsis of the results of the data retrieved from this cohort shows that a wide variety of underlying causes must be considered in patients complaining of adverse effects from dental materials. Besides dental and orofacial findings and allergies, these also include findings related to known general diseases or conditions and side effects from medications. Thus, special consultations that regularly deal with such complex patient cases are highly worthwhile for optimal diagnosis and medical counseling of these patients, preferably in close collaboration with other medical disciplines due to frequent links of the complaints to known general diseases and corresponding medications, because it is not easy for general dental practitioners themselves to investigate the specific background of a patient’s complaints. However, when facing a patient claiming adverse effects from dental materials, a general dental practitioner should take sufficient time for detailed anamnesis of the respective medical and dental history, intake of medications, and detailed dental and orofacial examinations before thinking of potential allergies that might be relevant to the complaints of the patient.
